# Tsallis *q*-Stat and the Evidence of Long-Range Interactions in Soil Temperature Dynamics

**DOI:** 10.3390/e23070909

**Published:** 2021-07-17

**Authors:** Babalola O. Ogunsua, John A. Laoye

**Affiliations:** 1 Key Laboratory for Middle Atmosphere and Global Environmental Monitoring (LAGEO), Institute of Atmospheric Physics (IAP), Chinese Academy of Sciences (CAS), Beijing 100864, China; 2 Department of Physics, Olabisi Onabanjo University, Ago-Iwoye 120107, Ogun State, Nigeria; bidemi.laoye@oouagoiwoye.edu.ng

**Keywords:** soil temperature, soil thermal diffusivity, *q*-Gaussian, non-extensive statistical mechanics, superstatistics

## Abstract

The complexities in the variations of soil temperature and thermal diffusion poses a physical problem that requires more understanding. The quest for a better understanding of the complexities of soil temperature variation has prompted the study of the *q*-statistics in the soil temperature variation with the view of understanding the underlying dynamics of the temperature variation and thermal diffusivity of the soil. In this work, the values of Tsallis stationary state *q* index known as *q*-stat were computed from soil temperature measured at different stations in Nigeria. The intrinsic variations of the soil temperature were derived from the soil temperature time series by detrending method to extract the influences of other types of variations from the atmosphere. The detrended soil temperature data sets were further analysed to fit the *q*-Gaussian model. Our results show that our datasets fit into the Tsallis Gaussian distributions with lower values of *q*-stat during rainy season and around the wet soil regions of Nigeria and the values of *q*-stat obtained for monthly data sets were mostly in the range 1.2≤q≤2.9 for all stations, with very few values *q* closer to 1.2 for a few stations in the wet season. The distributions obtained from the detrended soil temperature data were mostly found to belong to the class of asymmetric *q*-Gaussians. The ability of the soil temperature data sets to fit into *q*-Gaussians might be due and the non-extensive statistical nature of the system and (or) consequently due to the presence of superstatistics. The possible mechanisms responsible this behaviour was further discussed.

## 1. Introduction

The complexities in soil thermal diffusion and the complex thermal interaction between the soil and the atmosphere could be responsible for the sporadic changes in the soil temperature measurements. Different factors are responsible for the variations in the soil temperature and these factors can contribute greatly to the complex variations of the soil temperature [1,2,3]. Dynamical complexity, a phenomenon which has been attributed to the dynamics of the atmospheric parameters, have been studied and characterised in different ways [4,5,6]. Many researchers have attributed the complexity of the atmosphere as contributing factors to the complexity of soil temperature variations [7,8,9]. However, the underlining dynamics of both systems are different and as such, the intrinsic complex dynamics of the soil temperature propagation and the various factors affecting the thermal diffusivity of the soil are the major determinants of its internal temperature dynamics. The complexities in soil dynamical processes in aspects such as the soil hydrology have been evaluated using the entropic approach and it has been able to reveal the variations of soil dynamics resulting from varying hydrological processes [10,11], which usually affects the temperature variation [1]. The other intrinsic factors affecting the soil thermal diffusivity include soil vegetation cover [1,3,8,12], soil colour, soil texture and soil organic carbon [1,3,7,8,12].

As described earlier, most atmospheric temperature systems are easily characterized by dynamical complexity. This framework is based on the study of complexity in these systems considering the Tsallis statistical distribution. Since statistical distributions are particularly important in the understanding the complexity of a dynamical system. The dynamical complexity of atmospheric and many environmental systems are mainly characterized by superstatistics, which can be seen as a generalization of many other distributions that may also characterize the atmospheric systems. For example, the symmetric *q*-Gaussian can be easily derived into a superstatistical distribution [13,14,15]. Superstatistics can describe fluctuations in natural systems that follows from a χ^2^- distribution like the *q*-Gaussian [14,16]. This explains the reason why heavy tailed distributions such as the atmospheric temperature and rainfall has been found to assume superstatistical characteristics [17,18]. Besides the symmetrical *q*-Gaussian, the superstatistical distributions can also be derived from the log-normal distributions [14], which have been found to characterize the atmospheric humidity and precipitation [19]. Relating the distributions describing natural systems such as the atmosphere to the presence of superstatistics could be a good reason for the possible presence of *q*-Gaussians. This because, like the ability to derive the superstatistics from *q*-Gaussians, the *q*-Gaussians can also be derived from super statistics. However, the asymmetric *q*-Gaussian cannot be easily derived from superstatistical distributions, as the processes can be extremely complicated.

Alternatively, *q*-Gaussians can be derived from either Tsallis entropy or gamma distributions [20,21]. Particularly, gamma distributions allow for the formulation of asymmetric *q*-Gaussians. An extensive derivation of asymmetric *q*-Gaussian from gamma distributions can be found in Budini (2015) where he Generally classified the asymmetric *q*-Gaussians [21]. In this investigation, our interest in the consideration of *q*-Gaussians stems from the fact that gamma distributions have been observed in satellite rain rate measurements from tropical regions [22].

In this work, we have investigated the complexity of soil temperature and thermal diffusivity dynamics for a better understanding of the statistical mechanics of its variation. An attempt is made to investigate the dynamical processes in the soil temperature variation by considering the Tsallis formalism for *q*-Gaussian distribution, as we attempt to fit statistical distribution from the measured soil temperature data into Tsallis *q*-Gaussian. A good number of studies have considered the use of the Tsallis formalism in fitting experimental data into *q*-Gaussians [23,24,25,26,27,28,29,30]. To achieve this, we evaluate the non-extensive statistical mechanics in soil temperature time series measured from four different locations in Nigeria. The nonextensive statistical mechanics was investigated for soil temperature in this work with the following questions in consideration:

i. Does the Tsallis *q*-stat parameter vary seasonally for soil temperature?
ii. Is there a possible influence of changes in factors such as rainfall on the variations of the *q*-stat?
iii. Is there an influence of vegetational changes such as changes in vegetational cover soil texture and other intrinsic properties?

Our ability to answer these questions will not only shed more light on soil temperature dynamics but also provide a better understanding of the viability of the application of nonextensive statistical mechanics and Tsallis statistics to the characterization and modelling of soil temperature and soil thermal diffusivity in the future.

## 2. Tsallis Statistical Formalism and *q*-Gaussians

Tsallis statistics can be found in various natural systems, because as mentioned earlier, many experimental distributions obtained from natural systems can fit directly into Tsallis *q*-Gaussian [23,24,25,26,27]. The Boltzmann-Gibbs statistical mechanics is generally described by Gaussian distribution. However, the special class of statistical systems described by the Tsallis formalism are described by *q*-Gaussians [28,29,30,31,32,33].

As mentioned before the *q*-Gaussian can be derived from Tsallis entropy in which the Tsallis *q*-index plays a vital role. The Tsallis *q*-index which was introduced by Tsallis in 1988 provides the generalization of the Boltzmann-Gibbs statistical mechanics. The Tsallis entropy can be derived as follows [20,28,33]:

Given that the Shannon entropy from Boltzmann-Gibbs statistical Mechanics is:

(1) $$S_{sh}=k\underset{i}{\sum }P_{i}lnP_{i}$$

The non-extensive generalization of the Shannon entropy is therefore given as

(2) $$S_{q}=k\underset{i}{\sum }P_{i}ln_{q}P_{i}$$

$P_{i}$ is the probability density function of a discrete random variable $x_{i}$ such that

(3) $$\underset{i}{\sum }P_{i}=1$$

and the *q* logarithm $ln_{q}\left(x\right)$ is given as

(4) $$ln_{q}\left(x\right)=\left(x^{1-q}-1\right)/\left(1-q\right)$$

The parameter *q* is the Tsallis *q*-index, which varies for different systems based on the variations in their dynamical characteristics.

Putting expression (4) into (2) we arrive at Tsallis entropy in the form:

(5) $$S_{q}=k\frac{1}{1-q}\left(1-\overset{w}{\underset{i=1}{\sum }}p_{i}^{q}\right)$$

where p is the probability of each micro-configurations of the series, w is the total number of micro-configurations in the system and q is the Tsallis *q*-stat (which must be a real number). This Tsallis entropy expression is such that; for q=1 we obtain a special case of Tsallis entropy which results back into Boltzmann-Gibbs statistical mechanics where q≠1 results in non-extensive statistical mechanics.

Considering the continuous case for the random variable x, $S_{q}$ maximizes with the probability as follows:

(6) $$\overset{\infty }{\underset{-\infty }{\int }}P\left(x\right)dx=1$$

and

(7) $${\langle \left(x-\bar{x}\right)\rangle }_{q}=\overset{\infty }{\underset{-\infty }{\int }}\left(x-\bar{x}\right)P^{q}\left(x\right)dx=0$$

such that,

(8) $${\langle \left(x-\bar{x}\right)}^{2}\rangle {}_{q}=\overset{\infty }{\underset{-\infty }{\int }}{\left(x-\bar{x}\right)}^{2}P^{q}\left(x\right)dx=\sigma_{q}^{2}$$

This gives the Tsallis distribution in the form:

(9) $$P\left(x\right)\;\equiv {\frac{1}{Z}}_{q}{\left[1+\left(1-q\right)\beta_{q}{\left(x-\bar{x}\right)}^{2}\right]}^{1/\left(1-q\right)}$$

Combining Equations (11) and (14)

(10) $$\int P\left(x\right)dx={\frac{1}{Z}}_{q}\int {\left[1+\left(1-q\right)\beta_{q}{\left(x-\bar{x}\right)}^{2}\right]}^{\frac{1}{1-q}}=1$$

The normalization constant $Z_{q}$ is given by:

(11) $$Z_{q}=\int {\left[1+\left(1-q\right)\beta_{q}{\left(x-\bar{x}\right)}^{2}\right]}^{1/\left(1-q\right)}dx$$

Considering that the Lagrange multiplier β for (8) is given as:

$$\beta =1/\left(2\sigma_{q}^{2}Z_{q}^{q-1}\right),$$

we can show that:

(12) $$Z_{q}=C_{q}/\sqrt{\beta }$$

and

(13) $$C_{q}=\sqrt{\pi }\frac{\Gamma \left(\frac{1}{q-1}-\frac{1}{2}\right)}{\sqrt{q-1}\;\Gamma \left(\frac{1}{q-1}\right)}$$

From established theories [14,15], the *q*-Gaussian for a positive number β can be generalized as follows:

(14) $$G_{q}\left(\beta ;x\right)=\frac{\sqrt{\beta }}{C_{q}}e_{q}^{-\beta x^{2}}$$

where the normalizing constant $C_{q}$ for different values of *q* expressed as follows:

(15) $$C_{q}=\left\{\begin{array}{c} \frac{2\sqrt{\pi }\Gamma \left(\frac{1}{1-q}\right)}{\left(3-q\right)\sqrt{1-q}\Gamma \left(\frac{3-q}{2\left(1-q\right)}\right)},\;\;\;\;\;\;\;\;\;\;\;\;\;\;\;\;\;\;\;\;\;\;\;\;\;-\infty <q<1,\\ \sqrt{\pi },\;\;\;\;\;\;\;\;\;\;\;\;\;\;\;\;\;\;\;\;\;\;\;\;\;\;\;\;\;\;\;\;\;\;\;\;\;\;\;\;\;\;\;\;\;\;\;\;\;\;\;\;\;\;\;\;\;\;\;\;\;\;\;\;\;q=1,\;\;\\ \frac{\sqrt{\pi }\Gamma \left(\frac{3-q}{2\left(q-1\right)}\right)}{\sqrt{q-1}\Gamma \left(\frac{1}{q-1}\right)},\;\;\;\;\;\;\;\;\;\;\;\;\;\;\;\;\;\;\;\;\;\;\;\;\;\;\;\;\;\;\;\;\;\;\;\;\;\;\;\;\;\;\;\;\;1<q<3. \end{array}\right.$$

For q<1, the support of $G_{q}\left(\beta ;x\right)$ is compact since the density vanishes for $\left|x\right|>1/\sqrt{\left(1-q\right)}\beta$.

In the case of asymmetric *q*-Gaussians Budini (2015) introduced the asymmetric Poissonian *q*-Gaussian $G_{>1}^{p}(x|q,a,\beta )$ relating the function to Tsallis index *q*, the shape parameters α and $\alpha^{\prime }$ and the asymmetric parameter a [21].

The introduction of the Poissonian distribution in Ref. [21] is anchored on the introduction of two independent gamma functions $Y_{1}$ and $Y_{2}$, whose joint probability can be defined as:

(16) $$P\left(y_{1},\;y_{2}\right)=y_{1}^{\alpha -1}\;y_{2}^{\alpha^{\prime }-1}\frac{e^{-\left(y_{1}+y_{2}\right)/\theta }}{\theta^{\alpha +\alpha^{\prime }}\Gamma \left(\alpha \right)\Gamma \left(\alpha^{\prime }\right)}$$

It is assumed the scale θ the same, while α and $\alpha^{\prime }$ are the shape parameters of the functions $Y_{1}$ and $Y_{2}$ respectively.

An asymmetric *q*-Gaussian was derived as a function of functions $Y_{2}$ and $Y_{2}$

(17) $$X=f\left(Y_{1}\;,\;Y_{2}\right)$$

in which the random variables of X as follows:

(18) $$X=\frac{1}{\sqrt{\beta }}\frac{Y_{1}-Y_{2}}{2\sqrt{Y_{1}Y_{2}}}$$

The resulting distribution $P\left(x\right)$ for X, which is a function of $Y_{1}$ and $Y_{2}$, is given as

(19) $$P\left(x\right)=\frac{\sqrt{\beta }}{C_{\propto \propto^{\prime }}}{\left(\frac{1}{1+\beta x^{2}}\right)}^{\left(\propto +\propto^{\prime }+1\right)/2}{\left(\sqrt{1+\beta x^{2}}+\sqrt{\beta }x^{2}\;\right)}^{\propto -1}\times {\left(\sqrt{1+\beta x^{2}}-\sqrt{\beta }x^{2}\right)}^{\left(\propto^{\prime }-1\right)}$$

such that, the normalization constant is $C_{\alpha \alpha^{\prime }}$ given as $C_{\alpha \alpha^{\prime }}=2^{\alpha +\alpha^{\prime }-1}\Gamma \left(\alpha \right)\Gamma \left(\alpha \prime \right)/\Gamma \left(\alpha +\alpha^{\prime }\right)$.

The maximum value of this $P\left(x\right)$ above can be given as

(20) $$x_{M}=\frac{\alpha -\alpha^{\prime }}{\sqrt{\left(1+2\alpha \right)\left(1+2\alpha^{\prime }\right)\beta }}$$

In which the asymmetry condition that in the limit $\sqrt{\beta }x\gg 1$, the power law behaviour emerges as,

(21) $$\underset{x\to \infty }{\lim}P\left(x\right)\;\approx \;\frac{\sqrt{\beta }}{C_{\alpha \alpha^{\prime }}}2^{\alpha -\alpha^{\prime }}{\left(\frac{1}{\sqrt{\beta }\;x}\right)}^{2\alpha^{\prime }+1}$$

such that, in the limit $\sqrt{\beta }x\ll -1$ we have

(22) $$\underset{x\to -\infty }{\lim}P\left(x\right)\approx \frac{\sqrt{\beta }}{C_{\alpha \alpha^{\prime }}}2^{\alpha^{\prime }-\alpha }\;{\left(\frac{1}{-\sqrt{\beta }\;x}\right)}^{2\alpha +1}\;$$

The derived function asymmetric Poissonian function $G_{>1}^{p}(x|q,a,\beta )$ can be written as follows [21]:

(23) $$P\left(x\right)=\frac{\sqrt{\beta }}{C_{\alpha \alpha^{\prime }}}{\left(\frac{1}{1+\beta x^{2}}\right)}^{\left(\alpha +\alpha^{\prime }+1\right)/2}{\left(\frac{\sqrt{1+\beta x^{2}}+\sqrt{\beta }x}{\sqrt{1+\beta x^{2}}-\sqrt{\beta }x}\right)}^{\left(\alpha -\alpha^{\prime }\right)/2}$$

such that, the normalization constant is $C_{\alpha \alpha^{\prime }}$ given as $C_{\alpha \alpha^{\prime }}=2^{\alpha +\alpha^{\prime }-1}\Gamma \left(\alpha \right)\Gamma \left(\alpha \prime \right)/\Gamma \left(\alpha +\alpha^{\prime }\right)$.

The Tsallis *q*-index is related to the shape parameters α and $\alpha^{\prime }$ as follows:

(24) $$q=1-{\left[\frac{\alpha +\alpha^{\prime }}{2}-1\right]}^{-1},a=\frac{\alpha -\alpha^{\prime }}{2}$$

where $\alpha \neq \alpha^{\prime }$ in this asymmetric case. However, if$\;\alpha =\alpha^{\prime }$ and a=0, we have the symmetric case and Equation (7) reduces to

(25) $$P\left(x\right)=\frac{\sqrt{\beta }}{C_{\alpha }}{\left(\frac{1}{1+\beta x^{2}}\right)}^{\alpha +(1/2)}$$

The normalization constant in the symmetric case transforms to $C_{\alpha }=2^{\left(2\alpha -1\right)}\Gamma^{2}\left(\alpha \right)/\Gamma \left(2\alpha \right)$.

Considering the background on asymmetric *q*-Gaussian given by Budini [21] and the presence of Gamma distributions in natural systems like precipitation [22], we therefore consider that the presence of asymmetric *q*-Gaussian in natural system such as soil temperature, which is controlled by various forcing mechanisms is hugely possible. In previous work, we have shown that other systems such as the ionosphere have demonstrated such phenomenon [27].

## 3. Data and Methods

In this work, the measured soil temperature data were obtained from the National Space Research and Development Agency (NASRDA) and Centre for Atmospheric Research (CAR) data depositories in Nigeria. The data sets were measured using the sensors deployed in different weather stations at different regions of Nigeria.

**Figure 1:**
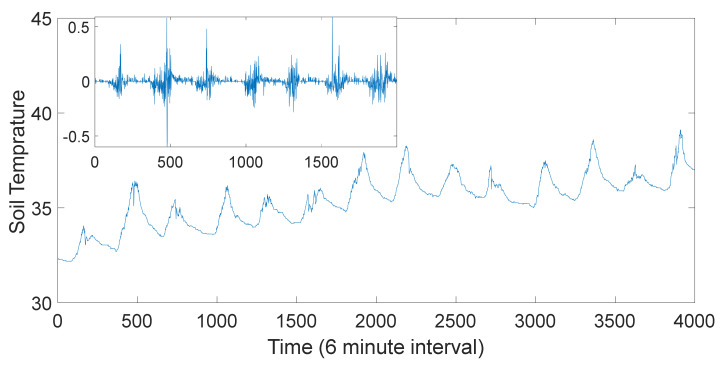
Time series plots. Outer Plot: Time plot showing the diurnal variations of the soil temperature time series. Inner Plot: Detrended time series for the soil temperature.

The measured temperature data characterizing the dynamics of soil temperature variation tend to possess sporadic variations in time. The time series measurement for soil temperature variation can be seen in Figure 1. The main diagram in Figure 1 is characterised by daily peaks due to diurnal variation, which dominates the intrinsic dynamics. Considering the purpose of our study, the internal variation of the soil temperature dynamics is required. Therefore, to obtain the intrinsic dynamics of the soil temperature we performed a detrending analysis on the soil temperature as to remove the diurnal effects follows [34]:

We let the measured time series be $x_{\left(t_{i}\right)}$ with a length v and the fixed-point average temperature values of $x_{fix\left(t_{j}\right)}$, where i=1,2,3……v represents the observed time series, and j=1,2,3…..v, such that the detrended or diurnal variation reduced time is given as

(26) $$x_{\left(t_{i}\right)}^{\prime }=x_{\left(t_{i}\right)}-x_{fix\left(t_{j}\right)}$$

where $i=1,2,3,\ldots .,\;j=mod\left(i,v\right),$ if $mod\left(j,v\right)\neq 0,$ and j=v if $d\left(j,v\right)=0$.

The detrending filter produces soil temperature time series $x_{\left(t_{i}\right)}^{\prime }$ as seen in the inner diagram in Figure 1. Further details on this process can be found in Ref. [34]. Similar analyses were carried on other time series to obtain the detrended time series before further analysis.

The datasets were further subjected to stationarity test, as this will ensure the sustainability of the datasets in modelling. The Dicky Fuller methods combined with the Kwiatkowski Philip Schmidt and Shinn (KPSS)-tests were conducted on the detrended soil temperature data sets [35,36,37].

The next step is to analyse our detrended time series data to obtain the probability distribution function (PDF) from which we obtain the values of the stationary state Tsallis *q*-index (*q*-stat).

Considering the theory of *q*-statistics [23,24,25,26], the probability distribution for the energy states $\left\{E_{i}\right\}$ is given by the expression

(27) $$P\left(E\right)\;\alpha {\left[1-\left(1-q\right)\beta E_{i}\right]}^{1/\left(1-q\right)}$$

and for the continuous state X in $\left\{X\right\},$ the probability density function becomes

(28) $$P\left(x\right)\;\alpha {\left[1-\left(1-q\right)\beta x^{2}\right]}^{1/\left(1-q\right)}$$

The expression $P\left(x\right)$ represents the probability of the dynamics of the microscopic elements $x_{j}$ in states $\left\{X\right\}$.

The stationary state *q*-index $q_{stat}$ can be derived based on the above theory of the from the probability distribution functions

(29) $$PDF\left(Z\right)\;\equiv A_{q}{\left[1+\left(1-q\right)\beta_{q}Z^{2}\right]}^{1/\left(1-q\right)}$$

where $PDF\;\left(Z\right)\equiv$ is the probability distribution with Z as the probability element, coefficients $A_{q}$, $\;\beta_{q}$ denote the normalization constants and $q=q_{stat}$ is the stationary state in this case for which q≤3.

Based on the *q*-Gaussian equation, we can estimate the value of q from time series $z_{i}$ with length i, that is, $Z:\left\{z_{1}z_{2}\ldots \ldots ,z_{i}\right\}$ estimate the optimum value of $q_{stat}$ from the series $z_{i}$ using the probability density function (PDF) above.

To fit the data to the probability density function, the values $X_{i}$ was divided into windows with width of small value Z with a $\left[Z_{min},\;Z_{max}\right]$ for which the frequency of Z; that fall within the Z intervals can be obtained. The normalized histogram for $PDF\left(Z\right)$ were obtained, such that, the probability density function $PDF\{P_{i}(Z_{i}$)} represents stationary PDF function.

The resulting fitting would give the corresponding value of Tsallis *q*-index. To obtain the power relation of this probability density function, we go further obtain the linear relationship between $log{\;}_{q}\left[P\left(Z\right)\right]$ and $Z^{2}$. This procedure was carried out for monthly soil temperature datasets measured from the following locations: Port-Harcourt (4.8156° N, 7.0498° E), Nsukka (6.8429° N, 7.3733° E), Makurdi (7.7322° N, 8.5391° E) and Yola (9.2035° N, 12.4954° E).

To evaluate for the degree of asymmetry in the *q*-Gaussian based on Ref. [21], we obtain the shape parameter ∝ by evaluating the gradient $S_{1}$ of linear fit of the normalized probability function H (Hnorm) plotted against the corresponding absolute value of |x| based on (21). Similarly, we obtain the shape parameter $\propto^{\prime }$ by evaluating the gradient $S_{2}$ of linear fit of the normalized probability function H (Hnorm) plotted against the corresponding absolute value of |−x| based in (22). The coefficient of asymmetry a, which can be obtained from the shape parameters ∝ and $\propto^{\prime }$ are related to the corresponding slopes as follows:

Recall that,

$$a=\left(\propto -\propto^{\prime }\right)/2$$

where $(\propto ,\propto^{\prime })\in A$ such that

(30) $$A\left(S\right)↔\left\{\begin{array}{c} \propto \approx \left(S_{1}-1\right)/2\\ \propto^{\prime }\approx \left(S_{2}-1\right)/2 \end{array}\right.$$

Therefore, the asymmetric parameter a is related to the gradients as follows:

(31) $$a≅\left(\left(S_{1}-S_{2}\right)\right)/4$$

The values of the asymmetric parameters were evaluated from the detrended soil temperature measured for different months of the year, based on this procedure.

## 4. Results and Discussion

In this work, the soil temperature time series was analysed to understand the statistical mechanics of the thermal diffusion processes and the energy transfer dynamics in the soil. The test for stationarity shows that the data sets are stationary. The Augmented Dickey Fuller (ADF) test for the data sets gave *p* values exceedingly less than 0.01, which gives us the confidence to reject the null hypothesis and classify the data sets as stationary and the KPSS test showed unity for all the values. Both cases of test results from the ADF test and the KPSS test show that we can reject the null hypothesis that the soil temperature data is stationary [35,36,37,38]. The stationarity of the data implies that our time series data can preserve the *q*-Gaussian model.

The monthly Tsallis *q*-stat was computed from monthly temperature data from different stations in Nigeria. This was carried out by dividing the annual soil temperature data into monthly segments and each of the segments was fitted into *q*-Gaussian. The values of *q*-stat obtained showed that the detrended soil temperature data generally satisfy the non-extensive statistical mechanics, as the *q*-index values were greater than 1 in all cases. The values of *q*-stat obtained from the *q*-Gaussian were in the range 1.2≤q≤2.9 (See Table 1, Table 2, Table 3 and Table 4). The observed ability of the atmospheric temperature data to fit the *q*-Gaussian might also be a consequence of the presence of superstatistics. As mentioned earlier, physical systems that exhibit non-extensive statistical mechanics have been linked with superstatistics [13,14,33].

**Figure 2:**
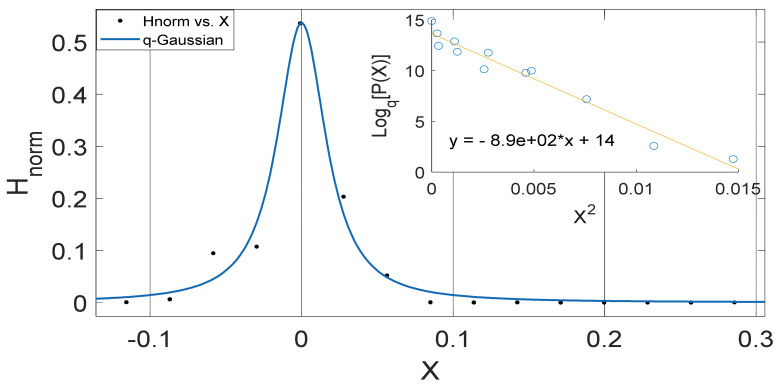
Outer Plot: Asymmetric Annual *q*-Gaussian for soil Temperature data measured in February 2009 Port-Harcourt. Inner Plot: Linear fit for the values of Log_q_ [P(Z)] vs. Z^2^ at q=1.867±0.592 fixed at bound.

**Figure 3:**
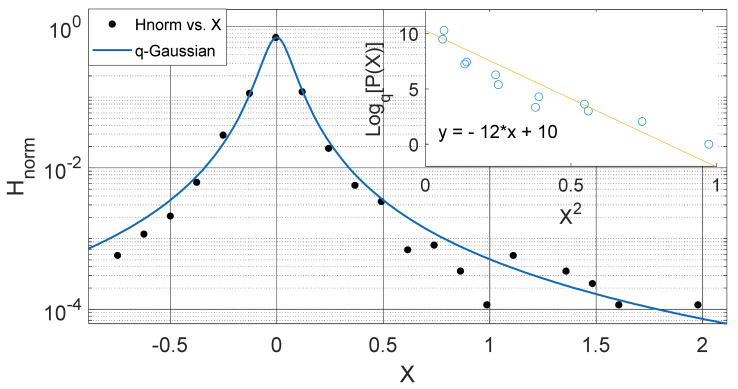
Outer Plot: Asymmetric Annual *q*-Gaussian for soil Temperature data measured in September 2009 at Port-Harcourt. Inner Plot: Linear fit for the values of Log_q_ [P(Z)] vs. Z^2^ at q=1.711±0.088.

**Figure 4:**
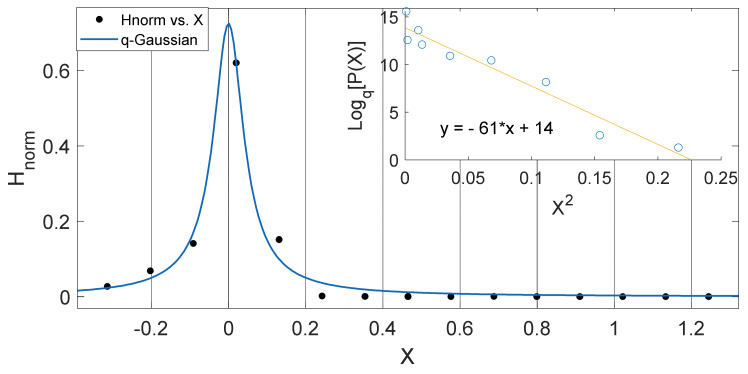
Outer Plot: Asymmetric Annual *q*-Gaussian for soil Temperature data measured in February 2011 at Nsukka, with high degree of Skewness. Inner Plot: Linear fit for the values of Log_q_ [P(Z)] vs. Z^2^ at q=2.148±0.63.

**Figure 5:**
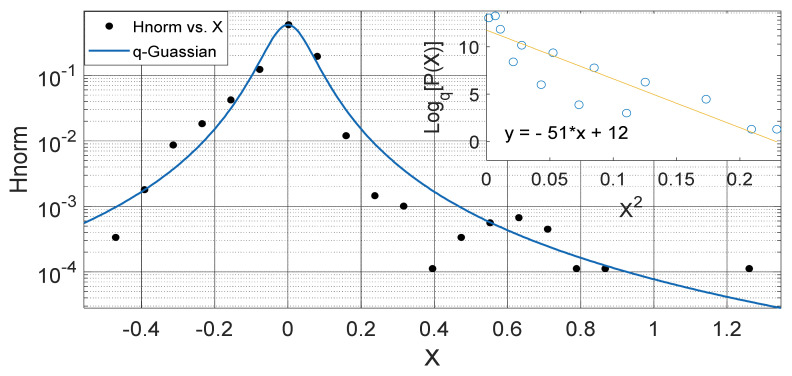
Outer Plot: Asymmetric Annual *q*-Gaussian for soil Temperature data measured in June 2011 at Nsukka, with high degree of Skewness. Inner Plot: Linear fit for the values of Log_q_ [P(Z)] vs. Z^2^ at q=2.045±0.234.

**Figure 6:**
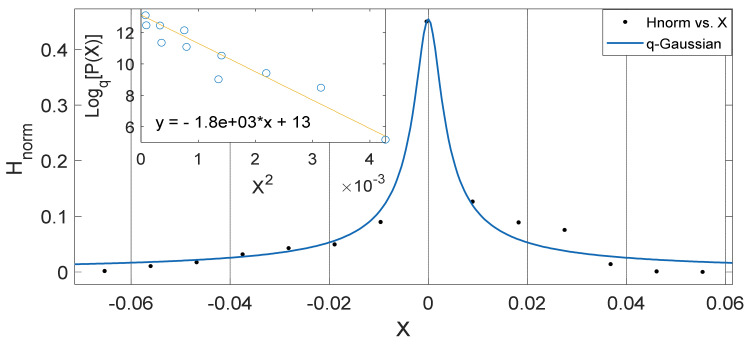
Outer Plot: Asymmetric Annual *q*-Gaussian for soil Temperature data measured in Jan 2009 at Makurdi, with high degree of Skewness. Inner Plot: Linear fit for the values of Log_q_ [P(Z)] vs. Z^2^ at q=2.892.

**Figure 7:**
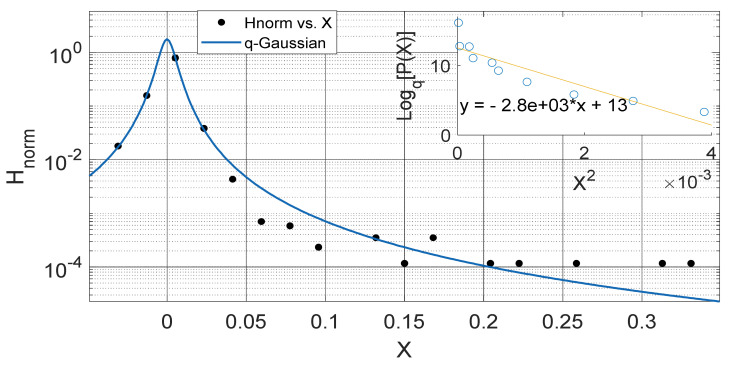
Outer Plot: Asymmetric Annual *q*-Gaussian for soil Temperature data measured in June 2009 at Makurdi, with high degree of Skewness. Inner Plot: Linear fit for the values of Log_q_ [P(Z)] vs. Z^2^ at q=1.744±0.178.

**Figure 8:**
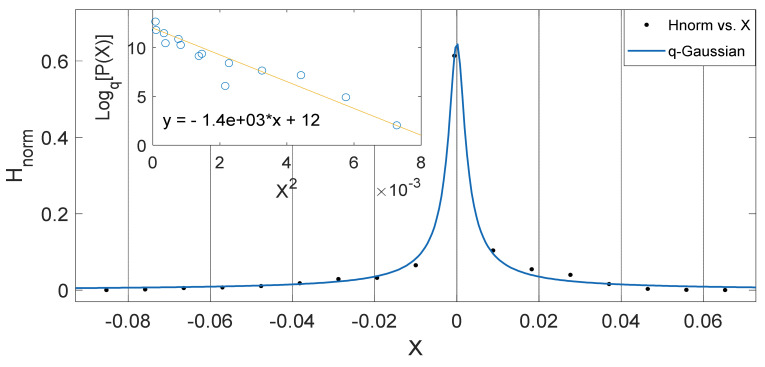
Outer Plot: Asymmetric Annual *q*-Gaussian for soil Temperature data measured in April at Yola, with high degree of Skewness. Inner Plot: Linear fit for the values of Log_q_ [P(Z)] vs. Z^2^ at q=2.606 fixed at bound.

**Figure 9:**
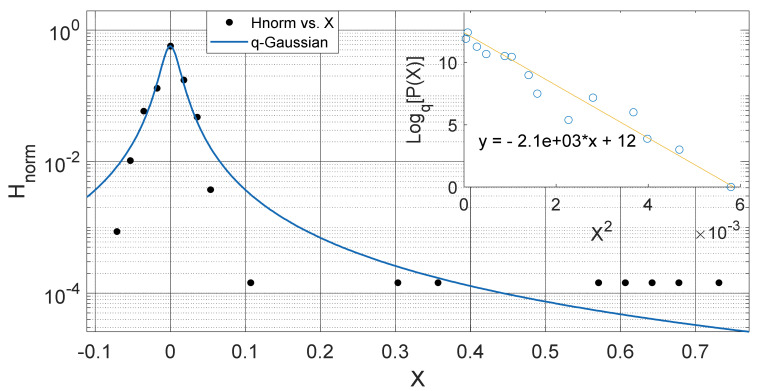
Outer Plot: Asymmetric Annual *q*-Gaussian for soil temperature data measured in December 2011 at Yola, with high degree of Skewness. Inner Plot: Linear fit for the values of Log_q_ [P(Z)] vs. Z^2^ at q=1.661±0.095 fixed at bound.

These observed results imply that the intrinsic properties of soil in different locations such as the thermal diffusivity, moisture variation, changes in vegetational cover might be responsible for the changes in the soil temperature dynamics and the variation in *q*-stat, which are found to be in a particular range for different locations. (See Figure 2, Figure 3, Figure 4, Figure 5, Figure 6, Figure 7, Figure 8 and Figure 9).

We found that the measured soil temperature data sets fit into *q*-Gaussians with *q*-stat values ranging from 1.2≤q≤2.8. However, it is possible that due to the system reorganization between the rainy season and the dry season, the soil temperature in the savannah region or in the dry season (for southern region) were mostly found to fit into the *q*-Gaussian model when the data sets were binned into distributions with larger width or class intervals. The distributions were found to assume *q*-Gaussian more accurately with less noise with larger class intervals during dry season. For instance, we found that for Yola and Makurdi stations, when the soil temperature time series distributions are subdivided into smaller class intervals, the resulting distribution could only fit into the *q*-Gaussian model from the month of May and June, respectively, and cannot fit into the *q*-Gaussian model after the rainy season from November (see Table 3 and Table 4). The observed possibility of smaller range classes, which allows for a distribution with larger number of bins in the wet season might be because of the presence of rain and high humidity in the raining season, modifying the micro changes in temperature variation. The raining season in Nigeria varies for different locations with longer rainy seasons in the south and shorter rainy seasons in the northern part of Nigeria. A good number of investigations have established that the soil water content is a strong determinant factor in soil temperature regulation [1,10].

The variations in soil texture for different locations might be another factor affecting the nature of the distributions. The soil texture is one of the characteristics of the soil that strongly affects the soil thermal diffusivity [2,7]. The soil structure in the southern part of Guinee savannah where Yola and Makurdi measuring stations are located which is also known as derived Savannah region [39] is mostly characterized by combination of loosely packed aeolian sandy soil and rocky (quartz) sediments, with high silica content [40]. The thermal conduction of soils with high silica can be highly consistent with minimal changes in temperature [7]. The thermal diffusivity of loosely packed soil structure in some areas of the Savannah may also affect the thermal conduction, as conduction is lower in loosely packed soil. As a result, the micro-changes soil temperature occurs in lower magnitude, due to high degree of aeration and conduction gaps resulting from the characteristically large pores found in the soil structure in this region. With less rain in the Savannah region the soil temperature tends to be more consistent. Also, the temperature could be more consistent due to the soil texture. A combination of these characteristics might be responsible for the near zero micro-changes in the soil temperature, hence, the small distributions in class intervals and larger value of *q*-Gaussian. This characterizes the dynamics of soil temperature in arid/Savannah region and possibly other regions with less rainfall and soil moisture differently, compared to other regions with a higher soil humidity and soil moisture content differently from seasons with rainfall in the same region. This is because the presence of moisture could greatly increase the soil thermal conductivity.

The values of *q* were suspected to change with the weather conditions, this might be responsible for the variations in *q* values especially for lower values of *q* below 1.3 and sometimes close to unity close to wet season in some locations. The lower values of *q*-stat being recorded in the wet season might be due the situation where the conductivity of the soil varies due to the moisture content. The atmosphere and other environmental systems have been characterized with a mixture of both chaotic and stochastic variations and sometimes with high degrees of freedom. These variations usually result in radical changes in the dynamical component of these environmental systems [41].

**Figure 10:**
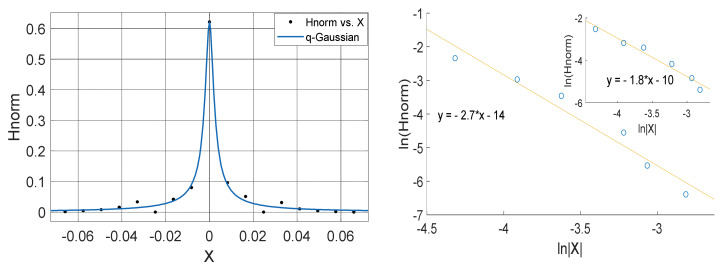
Left Panel: *q*-Gaussian for atmospheric temperature measured in Yola in February 2012 (Left Panel). Right Panel: Linear fit of $ln\left(Hnorm\right)$ vs. $ln\left|X\right|$ for positive values of X (outer plot) and negative values of X (inner plot); with the asymmetry a computed from the slopes in which a=−0.225.

**Figure 11:**
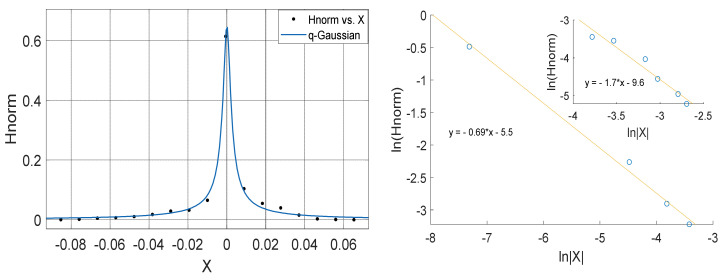
Left Panel: *q*-Gaussian for atmospheric temperature measured in Yola in April 2012. Right Panel: Linear fit of $ln\left(Hnorm\right)$ vs. $ln\left|X\right|$ for positive values of X (outer plot) and negative values of X (inner plot); with the asymmetry a computed from the slopes in which a=0.2525.

**Figure 12:**
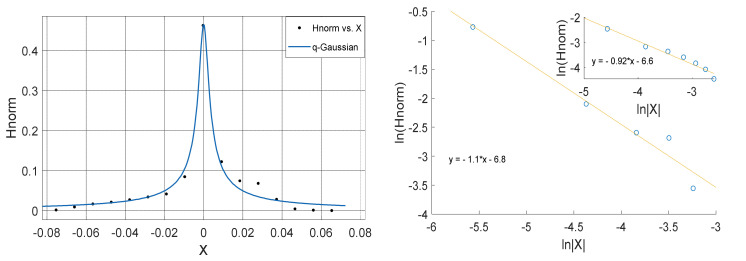
Left Panel: *q*-Gaussian for atmospheric temperature measured in Makurdi in February 2009. Right Panel: Linear fit of $ln\left(Hnorm\right)$ vs$\;ln\left|X\right|$ for positive values of X (outer plot) and negative values of X (inner plot); with the asymmetry a computed from the slopes in which a=−0.045.

**Figure 13:**
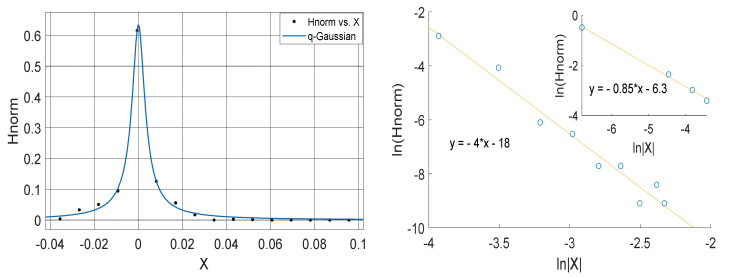
Left Panel: *q*-Gaussian for atmospheric temperature measured in Makurdi in July 2009. Right Panel: Linear fit of $ln\left(Hnorm\right)$ vs. $ln\left|X\right|$ for positive values of X (outer plot) and negative values of X (inner plot); with the asymmetry a computed from the slopes in which a=−0.7875.

Another interesting aspect of our results show that the *q*-Gaussians fitted from the soil temperature data classified under the extended group of asymmetric *q*-Gaussians (see Figure 2, Figure 3, Figure 4, Figure 5, Figure 6, Figure 7, Figure 8, Figure 9, Figure 10, Figure 11, Figure 12 and Figure 13), as derived in earlier (see also Ref. [21]). These asymmetric properties could be associated with the seasonal variations in energy transfer between the air and soil interface. The symmetrical changes in the *q*-Gaussian distributions obtained from the soil temperature data sets can be associated with the inconsistencies in the atmosphere–soil energy transfer interface. However, this asymmetric property might not be totally linked to changes in location as it could be found in the southern region for both Nsukka and Port-Harcourt and toward the northern region at Makurdi (see Figure 2, Figure 3, Figure 4, Figure 5, Figure 6 and Figure 7). However, the asymmetry in the *q*-Gaussian found at Yola towards the northern/middle belt region of Nigeria appeared to be lower compared to the *q*-Gaussians fitted for the other three locations (see Figure 8 and Figure 9). These inconsistencies may range from the inconsistencies due to vegetational cover, soil carbon and other parameters that could affect the thermal diffusivity of the soil from one location to the other. The effect of these varying factors on the soil–atmospheric energy transfer could be responsible for the asymmetric properties of soil temperature *q*-Gaussian.

The *q*-Gaussian produced from smaller distribution and their corresponding linear plots of $ln\left(Hnorm\right)$ vs. $ln\left|X\right|$ are shown in Figure 10, Figure 11, Figure 12 and Figure 13. The *q*-Gaussians are clearly defined with wider class intervals, particularly for savannah regions and dry seasons, as discussed earlier. These distributions tend exhibit varying degrees of asymmetry as observed from the *q*-Gaussians in the left panels of Figure 10, Figure 11, Figure 12 and Figure 13 and as shown in the previously presented figures. The slope of the linear plots of $ln\left(Hnorm\right)$ vs $ln\left|X\right|$ computed to evaluate the asymmetry parameters of the *q*-Gaussians (See Figure 10, Figure 11, Figure 12 and Figure 13 right panels). The values of asymmetry a tends to vary with season, as the values were lower in month of February, which falls within the dry season in this region, when the *q*-Gaussians were closer to symmetry. However, the values of asymmetry a were higher in the months of April at Yola and July at Makurdi which fall within early and deep rainy season in this region, respectively.

The asymmetry holds, since all values of a are greater than zero, that is, where a>0, then all the probability distributions presented here are asymmetrical. However, the degree of asymmetry becomes higher with higher values of $\left|a\right|$. In the cases where the values of $\left|a\right|\;\to \;0$ for a given probability distribution, then such a distribution tends towards symmetry.

The asymmetric parameters computed from the time series showed that the distributions obtained from detrended soil temperature were found to be more symmetrical in the dry season month, especially in the months of January and February with higher degrees of asymmetry during the rainy season from April, particularly for Savannah regions, where there is a clear distinction between the rainy season and dry season (see Figure 10, Figure 11, Figure 12 and Figure 13). These observations demonstrate the possible effect of humidity and rainfall as one of the possible driving forces responsible for changes in symmetrical positions of the *q*-Gaussian distributions. The shift in the symmetry shows that for different seasons the soil thermal system undergoes self-reorganisation. This changes in the symmetry during the rainy season can be attributed to the direct influence of the rainfall, which is a major atmospheric driver influencing the soil moisture content and hence the temperature regulation [10,41].

## 5. Conclusions

In this work, the Tsallis non-extensive statistical mechanics was investigated in soil temperature dynamics to obtain the stationary *q*-index for soil temperature measured from different regions of Nigeria. This was carried out by fitting the detrended soil temperature data into *q*-Gaussian. The analysis of soil temperature to obtain the Tsallis *q*-stat showed that virtually all distributions fit into the *q*-Gaussian model exhibiting the possible presence of non-extensive statistical mechanics. The ability of the detrended soil temperature time series to fit into Gaussian distribution demonstrates the possibility of representing the soil temperature with non-extensive statistical mechanics. It could also be because of the superstatistical nature of atmospheric systems, which are usually controlled by precipitation. As mentioned earlier, most of the physical systems that are characterized by non-extensive statistical mechanics usually fit into superstatistics. It was observed that the values of Tsallis *q*-stat varies with changes in the geographical belts of Nigeria as the values of *q*-stat varies increases towards the southern part of Nigeria. The statistical variations of soil temperature show that the monthly soil temperature distributions are dependent on seasonal variation, particularly considering the varying symmetry of the distributions. The asymmetry in the *q*-Gaussian, which varies seasonally has been attributed to the seasonal energy variations. The obtained result from this work shows that the Tsallis statistical mechanics can be used to determine and characterise the properties of the soil temperature and thermal diffusivity dynamics. Based on our observations, we infer that there is possible self-reorganization and dynamical forcing due to seasonal dynamical variations in the soil thermal diffusion. However, there is need for further investigations on the non-extensive dynamical properties of soil temperature for clearer understanding the drivers responsible for these variations in the future.

## Figures and Tables

**Table 1:** Tsallis *q*-stat for soil temperature measured at Port-Harcourt in 2009.

Month	Tsallis *q*-Stat	SSE	RMSE	R-Square
January	2.934	0.011118	0.03052	0.9456
February	1.867	0.006541	0.02335	0.9771
March	1.320	0.000327	0.00452	0.9994
April	1.395	0.000014	0.00093	1.0000
May	2.016	0.002225	0.01179	0.9953
June	2.024	0.001031	0.00803	0.9982
July	1.593	0.000386	0.00491	0.9990
August	1.468	0.000346	0.00465	0.9995
September	1.599	0.000206	0.003589	0.9996
October	1.575	0.000301	0.004334	0.9994
November	1.417	0.000019	0.001083	1.0000
December	1.977	0.002105	0.011470	0.9927

**Table 2:** Tsallis *q*-stat for soil temperature measured at Nsukka in 2011(—incomplete data).

Month	Tsallis *q*-Stat	SSE	RMSE	R-Square
January	--------	---------	----------	---------
February	2.148	0.005725	0.02184	0.9843
March	1.738	0.001648	0.00931	0.9971
April	1.887	0.024250	0.03320	0.9510
May	1.701	0.002174	0.01043	0.9959
June	1.608	0.000377	0.00434	0.9993
July	1.587	0.004064	0.01426	0.9888
August	1.548	0.000086	0.00277	0.9999
September	1.653	0.000081	0.00202	0.9999
October	1.939	0.00103	0.00718	0.9977
November	1.709	0.016350	0.03691	0.9427
December	---------	----------	----------	--------

**Table 3:** Tsallis *q*-stat for soil temperature measured at Makurdi in 2009 (—incomplete data).

Month	Tsallis *q*-Stat	SSE	RMSE	R-Square
January	2.892	0.004172	0.01947	0.9718
February	2.870	0.002934	0.01502	0.9845
March	2.952	0.003258	0.01648	0.9809
April	1.796	0.000492	0.00555	0.9989
May	1.715	0.000626	0.00417	0.9985
June	1.744	0.001205	0.00709	0.9972
July	2.246	0.001027	0.00889	0.9966
August	1.837	0.000131	0.00286	0.9997
September	1.725	0.000024	0.00116	1.0000
October	1.652	0.000021	0.00097	1.0000
November	------	----------	---------	-------
December	------	----------	---------	-------

**Table 4:** Tsallis *q*-stat for soil temperature measured at Yola in 2012.

Month	Tsallis *q*-Stat	SSE	RMSE	R-Square
January	2.662	0.0007251	0.008119	0.9978
February	2.869	0.0040690	0.01705	0.9771
March	2.753	0.0014070	0.01131	0.9947
April	2.606	0.0012227	0.00936	0.9964
May	1.914	0.0000334	0.001741	1.0000
June	1.572	0.0000261	0.001278	1.0000
July	1.468	0.0000009	0.000287	1.0000
August	1.326	0.0000007	0.000216	1.0000
September	1.60	0.0000016	0.000266	1.0000
October	1.702	0.0000560	0.001764	0.9999
November	2.507	0.0015540	0.011890	0.9951
December	1.661	0.0001864	0.002376	0.9997

## Data Availability

The data used in this work can be accessed through: https://carnasrda.com/necop/.
